# Supplementary material for: Nano-Confined Tin Oxide in Carbon Nanotube Electrodes via Electrostatic Spray Deposition for Lithium-Ion Batteries
Source: Materials (Basel). 2022 Dec 19;15(24):9086. doi: 10.3390/ma15249086 (PMC9786169; doi:10.3390/ma15249086)
Supplement: Supplementary file 1 [file materials-15-09086-s001.zip › materials-2060331-supplementary.pdf]

Supplementary Information

Nano-Confined Tin Oxide in Carbon Nanotube Electrodes via Electrostatic Spray Deposition for Lithium-Ion Batteries

Alexandra Henriques[a1], Amin Rabiei Baboukani[a1], Borzooye Jafarizadeh[1], Azmal Huda Chowdhury[1], and Chunlei Wang[1,2*]

[1]Department of Mechanical and Materials Engineering, Florida International University, Miami, FL 33174, USA

[2]Center for the Study of Matter at Extreme Conditions (CeSMEC), Florida International University, Miami, FL 33199, USA

*wangc@fiu.edu (C. Wang)

[a]A.H. and A.R.B. have equal contributions in this paper.

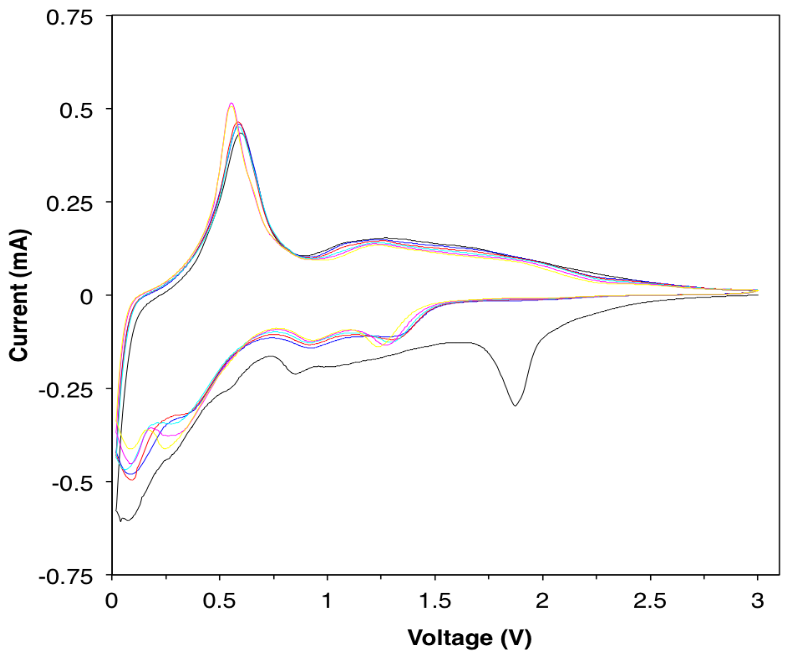

Figure S1: Cyclic voltammetry of SnO_2 alone in the voltage window of 0.01-3.0 V

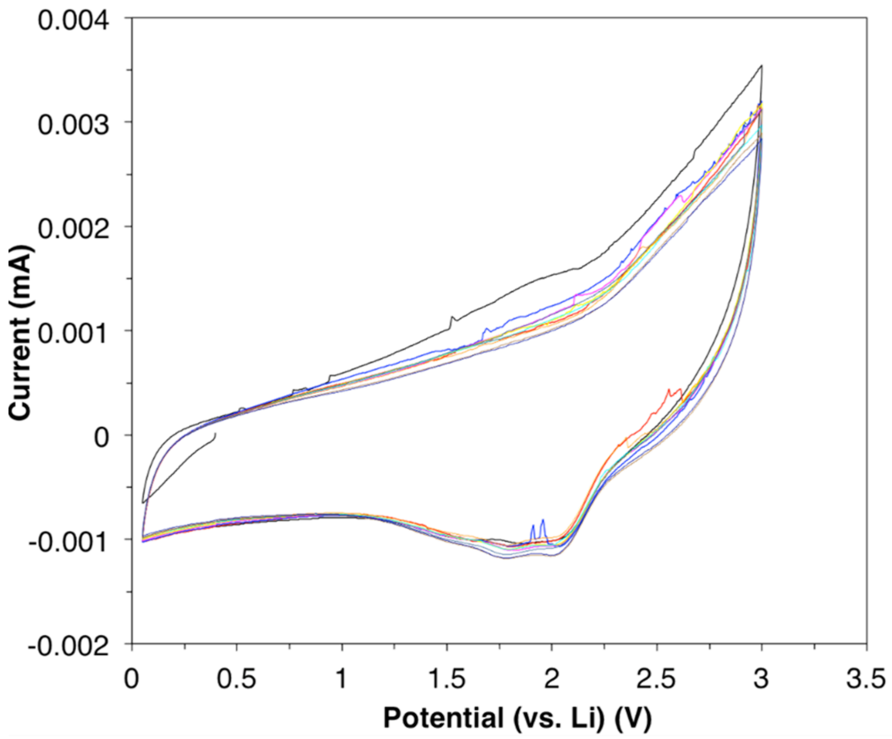

Figure S2: Cyclic voltammetry of CNT alone in the voltage window of 0.01-3.0 V

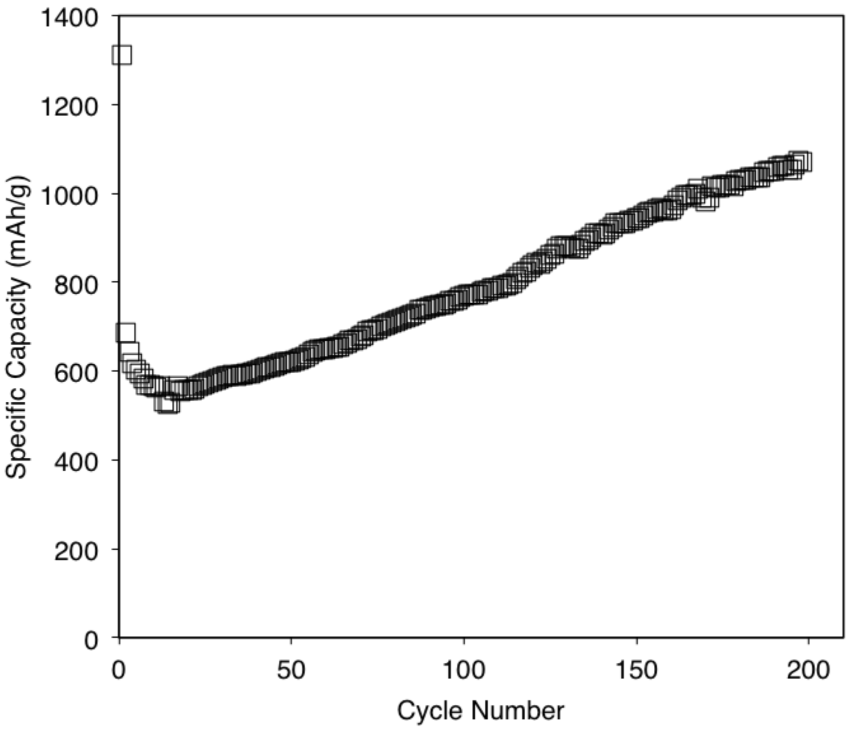

Figure S3: Cycle performance of 20% SnO_2-in-CNT for 200 cycles at 100 $mAhg^{-1}$
